# Update on ventilator-associated pneumonia

**DOI:** 10.12688/f1000research.12222.1

**Published:** 2017-11-29

**Authors:** Jean-Francois Timsit, Wafa Esaied, Mathilde Neuville, Lila Bouadma, Bruno Mourvillier

**Affiliations:** 1 IAME, Inserm U1137, Paris Diderot University, Paris, F75018, France; 2 Medical and Infectious Diseases Intensive Care Unit, AP-HP, Bichat University Hospital, Paris, France

**Keywords:** ventilator-associated pneumonia, VAP, nosocomial infection, antimicrobials

## Abstract

Ventilator-associated pneumonia (VAP) is the most frequent life-threatening nosocomial infection in intensive care units. The diagnostic is difficult because radiological and clinical signs are inaccurate and could be associated with various respiratory diseases. The concept of infection-related ventilator-associated complication has been proposed as a surrogate of VAP to be used as a benchmark indicator of quality of care. Indeed, bundles of prevention measures are effective in decreasing the VAP rate. In case of VAP suspicion, respiratory secretions must be collected for bacteriological secretions before any new antimicrobials. Quantitative distal bacteriological exams may be preferable for a more reliable diagnosis and therefore a more appropriate use antimicrobials. To improve the prognosis, the treatment should be adequate as soon as possible but should avoid unnecessary broad-spectrum antimicrobials to limit antibiotic selection pressure. For empiric treatments, the selection of antimicrobials should consider the local prevalence of microorganisms along with their associated susceptibility profiles. Critically ill patients require high dosages of antimicrobials and more specifically continuous or prolonged infusions for beta-lactams. After patient stabilization, antimicrobials should be maintained for 7–8 days. The evaluation of VAP treatment based on 28-day mortality is being challenged by regulatory agencies, which are working on alternative surrogate endpoints and on trial design optimization.

## Introduction

Hospital-acquired pneumonia (HAP) is defined by an infection of the lung parenchyma that occurred at least 48 hours after hospital admission. Ventilator-associated pneumonia (VAP) develops in intensive care unit (ICU) patients mechanically ventilated for at least 48 hours ^1, 2^. In contrast, ventilator-associated tracheobronchitis (VAT) is characterized by signs of respiratory infection without new radiographic infiltrates in a patient mechanically ventilated for at least 48 hours ^3– 5^.

In the past 10 years, a great deal of progress has been made in understanding VAP. New concepts of infection-related ventilator-associated complications (IVACs) and ventilator-associated events (VAEs) have been proposed as outcome indicators for prevention strategies ^6^. In diagnostic strategies, criteria used to suspect a VAP have been challenged, as have optimal diagnostic tests used to confirm it ^7^. Traditional risk factors of VAP due to multidrug-resistant (MDR) bacteria (based on early-onset occurrence and previous antimicrobial therapy) are no longer sufficient. Proposed empirical therapy has been modified accordingly. The optimization of pharmacokinetic/pharmacodynamic parameters is now considered a key factor to ensure adequate and successful therapy. The use of adjunctive aerosolized therapy is also more and more debated. In addition, regulatory agencies are trying to find surrogate endpoints to replace 28-day mortality and to improve the design of randomized clinical trials in this field of investigation ^8^.

For VAP prevention, the concept of bundle of care was defined. It enabled great successes in VAP prevention; however, the insufficient compliance observed in clinical practice needs to be addressed in order to define easier-to-apply procedures.

This review aims to summarize the available knowledge on VAP, taking profit from the recent publication of North American ^7^ and European guidelines on VAP management and highlighting recent advances and remaining controversies of the new concepts.

## Epidemiology

VAP is the second most common nosocomial infection and the leading cause of death from nosocomial infections in critically ill patients ^9^. Its incidence ranges from 5% to 67% depending on case mix and the diagnostic criteria used ^10^, and the highest rates are in immunocompromised, surgical, and elderly patients. In the US, the incidence of VAP ranges from 2 to 16 episodes per 1,000 ventilator-days ^11^. The estimated risk of VAP is 1.5% per day and decreases to less than 0.5% per day after the 14th day of mechanical ventilation ^12^. VAP increases the duration of hospitalization by 7 days and health-care costs by approximately $40,000 USD ^13^.

In published studies, the crude mortality of patients with VAP is highly variable according to case mix and definitions used ^14– 16^. The definition of attributable VAP mortality is the percentage of deaths that would not have occurred in the absence of the infection. Recent studies have reappraised the impact of VAP on mortality ^17– 19^. Specifically, given that the risk of VAP is time-dependent, this could potentially result in a significant time-dependent bias because mortality and ICU discharge both act as competing endpoints. Indeed, the most recent studies reported an attributable mortality below 10% with surgical patients ^18^ whereas those with mid-range illness severity presented the highest associated risk ^18, 19^.

Late-onset VAP is often reported to be associated with higher mortality rates than early-onset VAP ^20– 22^. Using a multistate model, we confirmed that the attributable mortality for early-onset VAP (5.8%) was considerably lower than for late-onset VAP (10.6%) ^18^.

Most studies showed that VAP is usually due to aerobic *Enterobacteriaceae* (25%), *Staphylococcus aureus* (20%), *Pseudomonas aeruginosa* (20%), *Haemophilus influenza* (10%), and streptococci ^23^. MDR pathogens are more common among late-onset cases. Trouillet *et al*. ^24^ found that prior use of broad-spectrum antibiotics and mechanical ventilation of more than 7 days were independent risk factors of infection caused by MDR pathogens. However, more recent reports ^25– 29^ have identified similar rates of etiologies in patients with early- versus late-onset VAP. This may be related to the worldwide rise in MDR pathogens; it emphasizes that the local ICU ecology ^30^ is the most important risk factor for acquiring MDR pathogens, irrespective of the length of intubation. In early-onset pneumonia, the initial VAP severity—that is, the presence of sepsis or septic shock (odds ratio [OR] = 3.7)—and pneumonia that developed in a center with a prevalence of resistant pathogens greater than 25% were independently associated with the presence of resistant pathogens (OR = 11.3) ^26^.

## Risk factors of ventilator-associated pneumonia

VAP results from the microbial invasion of the normally sterile lower respiratory tract, which subsequently can overwhelm the host’s defense and establish infection. The major route for microbial invasion is microaspiration of oropharyngeal secretions contaminated by endogenous flora around the endotracheal tube cuff ^31^. VAP may also occur by other means ^2, 32^. In terms of potential reservoirs, it has been suggested that the stomach hosts bacteria that colonize the oropharynx. It has been postulated by some researchers that embolization into the alveoli during suctioning or bronchoscopy is caused by the colonization of the endotracheal tube with bacteria encased in a biofilm ^33^. Inhalation of pathogens from contaminated aerosols and direct inoculation are less common, and hematogenous spread from either infected intravascular catheters or bacterial translocation of the gastrointestinal tract lumen are rarer in occurrence.

Consequently, two groups of risk factors for VAP have been identified—namely ventilation-related factors (instrumentation of the airway with an endotracheal tube and subsequent microaspirations) and, less frequently, patient-related factors (for example, pre-existing pulmonary disease)—and only the former is accessible to prevention ( Table 1). As a result, VAP, unlike many other nosocomial infections, is difficult to prevent ^34^.

**Table 1.** Risk factors of ventilator-associated pneumonia.

Host-related risk factors	Intervention-related risk factors
Medical history and underlying illness Male gender Extreme age Prior central nervous system disorder Immunocompromised Acute underlying diseases Emergent surgery Neurosurgery Thoracic surgery Cardiac surgery Burns Re-intervention Acute severity factors Organ system failure index of at least 3 Acute renal failure Acute respiratory distress syndrome ECMO, intra-aortic support Ulcer disease	Peri-operative transfusion of blood products Duration of the mechanical ventilation Reintubation Supine head position in patients receiving enteral nutrition Antibiotic therapy ^a^ Enteral nutrition Absence of subglottic secretion drainage ^b^ Intra-hospital transports Continuous sedation, use of paralytic agents Nasogastric tubes Tracheostomy Frequent ventilator circuit changes Intracuff pressure of less than 20 cm H _2_O

Adapted from 2, 35– 38. ^a^Antibiotic therapy protects from early-onset pneumonia due to susceptible bacteria but is a risk factor for late-onset pneumonia due to more resistant organisms. ^b^Protective impact of subglottic secretion drainage is mainly demonstrated for cardiac surgery patients. ECMO, extra-corporeal membrane oxygenation.

## Prevention

**Figure 1.**
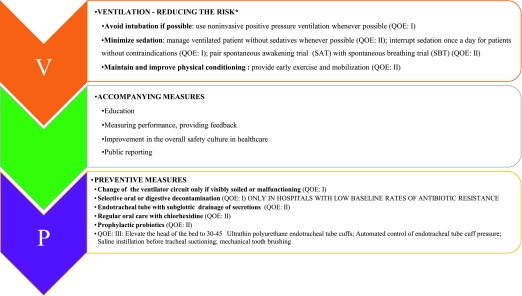
Preventive measures of ventilator-associated pneumonia. Adapted from 37, 39. QOE, quality of evidence.

First of all, reducing the exposure to risk factors for VAP is the most efficient way to prevent VAP onset ( Figure 1). Therefore, intubation should be avoided whenever possible, and strategies such as non-invasive positive-pressure ventilation, sedation, and weaning protocols should be used to replace or shorten mechanical ventilation. In contrast, recent data suggest that the timing of the tracheotomy does not significantly change VAP incidence ^40– 43^.

Patients at risk of VAP must be managed with a “bundle of preventive measures” ( Figure 1). Indeed, no single preventive strategy will efficiently prevent VAP. Bundles group together a small straightforward set of key interventions that are from evidence-based guidelines—generally three to five—and that are expected to result in a better outcome when performed collectively and reliably instead of individually. However, the ideal set of key preventive measures is unknown ^44^. Importantly, although studies demonstrated great success in reducing VAP rates using bundle of care in recent years ^44– 48^, meta-analyses showed that most of the preventive measures failed to demonstrate a sustained effect ^49^. This conclusion is in line with the absence of substantial improvement of VAP rates in the past decade ^50^. Figure 1 lists recommended preventive measures according to their level of evidence.

The sole preventive measures that positively impacted mortality are selective digestive decontamination (SDD) and selective oropharyngeal decontamination (SOD) ^51^. Compared with SOD, SDD was associated with a lower mortality, reduced length of stay, lower rates of ICU-acquired bacteremia and candidemia, and lower prevalence of rectal carriage of antibiotic-resistant Gram-negative bacteria but with a pronounced gradual increase in aminoglycoside-resistant Gram-negative bacteria ^52^. The main remaining question is the reproducibility of these results out of the Netherlands. Indeed, the antibiotic selection pressure induced by SOD or SDD may outweigh their benefits in countries with high levels of bacterial resistance.

Oral care with chlorhexidine is also debated. An updated meta-analysis focusing on double-blind studies in non-cardiac surgery patients showed that it had no impact on VAP rates or duration of mechanical ventilation or duration of ICU stay ^53^.

Ecological Effects of Decolonization Strategies in Intensive Care (RGNOSIS), a cluster-randomized study (ClinicalTrials.gov identifier NCT02208154) conducted in six European countries, is currently enrolling 10,800 patients into four arms: control, oral care with chlorhexidine, SOD, and SDD. The study’s new insights into these ongoing debates are awaited.

Many possible factors may explain why prevention measures did not result in reductions in mortality, duration of stay, or antibiotic consumption. First of all, the VAP definition may not be sufficiently accurate, especially when tested intervention could not be blinded. Second, in recent studies using modern statistics, the attributable mortality of VAP is only 3–4%, considerably smaller than previously reported ^19^. Both factors may induce a dramatic decrease of the power of the studies available.

Even if there is convincing evidence that specific interventions might prevent VAP, translating research into practice remains a challenge ( Figure 1). Two European surveys found that 37.0% of ICU physicians ^54^ and 22.3% of nurses ^55^ did not comply with the published recommendations for VAP prevention. Beyond the theoretical frame, a great deal of attention must be given to the factors that might facilitate a bundle implementation and allow a sustained compliance. An educational session alone, without an associated behavioral strategy, is unlikely to induce profound behavioral changes. It should be kept in mind that, to engage an individual in a particular behavior and improve compliance, we need to act on predisposing factors (knowledge, perceptions, and beliefs) to favor the access to new processes or technologies and to continually reinforce the behavior by feedback ^56, 57^.

## Diagnosis

### VAP, VAE, IVAC, and VAT: what do these abbreviations mean?

The diagnosis of VAP is traditionally based on clinical symptoms and radiographic criteria that require further bacteriological confirmation. However, it has been demonstrated that these criteria are inaccurate ^14, 58, 59^. Of note, VAP is now considered an indicator of performance in the US and some other countries. The National Healthcare Society Network reported a considerable decrease in the VAP incidence rate attributed to a multifaceted infection control program and its effective implementation ^60^. A 70% decrease of the incidence between 2006 and 2012 was reported by the Centers for Disease Control and Prevention (CDC). But in the same period, the Medicare Patient Safety Monitoring System reported an adjusted average annual change of 0% (95% confidence interval (CI) −0.05 to 0.07) in patients 65 years old or older ^50^. These findings emphasize a true discrepancy between rates reported in a quality monitoring program and rates observed in patients’ care program.

The discrepancies between results prompted the CDC to promote new objectives of the surveillance based on VAEs. Another important motivation of the CDC was to expand the purview of quality and safety surveillance to encompass multiple complications in mechanically ventilated patients instead of just pneumonia alone. 

**Figure 2.**
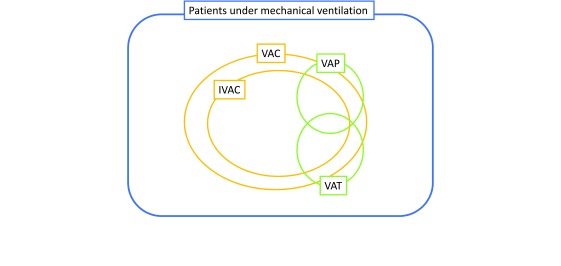
Ventilator-associated events, definitions, and nosology. *Ventilator-associated conditions (VACs)*: at least 2 calendar days of stable or decreasing daily minimum positive end-expiratory pressure (PEEP) or fraction of inspired oxygen (FiO _2_) followed by rise in PEEP of at least 3 cm H _2_O or rise in FiO _2_ of at least 20 points sustained for at least 2 days. *Infection-related ventilator-associated complications (IVACs)*: VAC plus: temperature of less than 36°C or more than 38°C OR white blood cell (WBC) count of not more than 4 or at least 12 × 10 ^3^ cells/mm ^3^ AND at least one new antibiotics continued for at least 4 days WITHIN 2 days of VAC onset EXCLUDING first 2 days on the ventilator. *Possible ventilator-associated pneumonia ****(VAP) (Centers for Disease Control and Prevention [CDC] definitions)*: IVAC plus: criterion 1: Positive culture meeting specific quantitative or semi-quantitative threshold; criterion 2: Purulent respiratory secretions AND identification of organisms NOT meeting the quantitative or semi-quantitative thresholds; criterion 3: Organisms identified from pleural fluid specimen, positive lung histopathology, and positive diagnostic test for Legionella species or selected respiratory viruses WITHIN 2 days of VAC onset EXCLUDING first 2 days on the ventilator. (The updated January 2017 definitions and comprehensive examples are detailed in the CDC National Healthcare Society Network website; https://www.cdc.gov/nhsn/pdfs/pscmanual/10-vae_final.pdf; accessed 23 October 2017.) *VAP*: radiographic criteria (new or progressive and persistent infiltrates or consolidation or cavitation); systemic criteria (temperature of less than 36°C or more than 38°C OR WBC count of not more than 4 or at least 12 × 10 ^3^ cells/mm ^3^); pulmonary criteria (at least one of the following: (1) new onset or increase of purulent aspirates and (2) worsening gas exchange). *Ventilator-associated tracheobronchitis (VAT)*: criteria for VAP but without radiographic criteria.

The VAE surveillance definition algorithm uses three new indicators: ventilator-associated complications (VACs), IVACs, and possible and probable VAP ^61^. VAC is the first step of VAE surveillance, with the aim of identifying any complication occurring in mechanically ventilated patients, regardless of the origin or mechanism. To meet the definition of VAC, a mechanically ventilated patient must have at least 2 days of stability or improvement of respiratory parameters—such as a stable or decreasing daily minimum positive end-expiratory pressure (PEEP) or fraction of inspired oxygen (FiO _2_)—followed by at least 2 days of worsened oxygenation (diagnosed by an increase of the daily minimum PEEP (at least 3 cm H _2_O) or FiO _2_ (at least 20%)). The concept of IVAC aims to identify the subgroup of VACs that are potentially related to infection. A VAC associated with an abnormal white blood cell count or a modified temperature becomes an IVAC if the initiation of a new antimicrobial agent is maintained for at least 4 days. With evidence of purulent respiratory secretions or positive results of microbiological tests performed on respiratory tract specimens or both, an IVAC becomes a possible VAP. All of these definitions are summarized in Figure 2.

The VAE concept uses objective criteria and their collection can be automated for systematic recording. The VAP definition is still widely discussed, and a recent study showed that applying the various diagnostic criteria to the same patient population resulted in large differences in the incidence of VAP (that is, from 4% to 42%) ^62^. Furthermore, even distal quantitative samples are not 100% reproducible ^63, 64^.

This new approach might overcome the inaccuracy of the VAP definition, facilitate its electronic assessment, and make inter-ICU comparisons more relevant. Second, the association between VAE and antibiotic consumption (considering VAC rates and not only IVAC) was a point in favor of using VAC rate as one indicator of ICU quality of care for antimicrobial stewardship programs ^12^. Of note, VAE has very low sensitivity and specificity in diagnosing VAP ^12^. In our experience, VAP accounted for only 14.5% of the VAC episodes and 27.6% of the IVAC episodes ^12^; in addition, not all IVAC episodes were related to a nosocomial infection.

Furthermore, radiological criteria are not taken into account, so that the IVAC definition includes VAT and VAP ( Figure 2). Although VAP and VAT are both associated with an increased duration of mechanical ventilation, VAP impact on ICU mortality is higher than that of VAT ^27^. Finally, embedding VAP in the larger definition of IVAC may hamper the understanding of VAP pathophysiology and thus its prevention improvement.

### Which bacteriological samples should be collected in case of suspicion of ventilator-associated pneumonia?

Great controversies persist about the bacteriological samples that should be used for diagnosing VAP. Of note, when bacteriological analyses are not immediately available, processing of a bacteriological specimen refrigerated after collection is a reliable alternative ^65^. Invasive techniques, such as bronchoalveolar lavage or protected specimen brush with quantitative culture, require qualified clinicians. Randomized studies that have evaluated their value as compared with proximal qualitative samples yielded contradictory results ^66– 68^. In one study with 413 patients ^67^, the invasive distal quantitative strategy was combined with an algorithm for treatment de-escalation and led to a significant increase in the number of antibiotic-free days at day 14 (5.0 ± 5.1 versus 2.2 ± 3.5) and day 28 (11.5 ± 9.0 versus 7.5 ± 7.6) in comparison with the strategy with non-invasive methods using qualitative cultures. In contrast, the Canadian Critical Care Trial Group reported no impact of distal quantitative samples on the day-28 antibiotic-free days or on survival ^66^. However, in that study, the research protocol may have facilitated appropriate discontinuation of antibiotics or targeted therapy in the two groups, thus minimizing the differences between them. Cohort studies confirmed the potential advantages of distal quantitative samples in narrowing antimicrobial therapy and limiting antibiotic selection pressure without adverse effects on mortality or length of stay ^69– 71^. Finally, an observational study in 89 patients with clinically suspected VAP and a negative quantitative bronchoalveolar lavage compared patients with early (within one day) and late antibiotic discontinuation. Early discontinuation was associated with a non-significant decrease in mortality and significantly lower risks of overall superinfections (22.5% versus 43%), respiratory superinfections (10% versus 29%), and superinfections due to MDR pathogens (7.5% versus 36%) ^72^.

Considering available literature, recent US guidelines recommend non-invasive sampling with semi-quantitative culture ^7^, whereas the European guidelines suggest obtaining distal samples with quantitative cultures to improve the accuracy of results ^9^. Despite this discrepancy, the two guidelines agreed that a bacteriological sample should be performed before any antibiotic treatment in order to reduce antibiotic exposure.

## Treatment of ventilator-associated pneumonia

**Figure 3.**
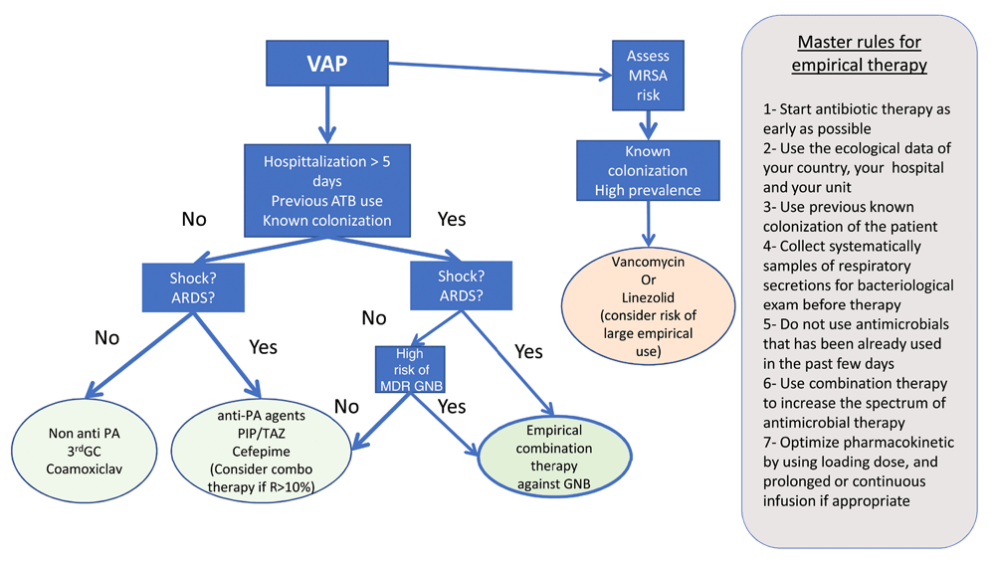
Proposed strategy for empirical therapy. *In areas with a risk of multidrug-resistant and carbapenemase-producing bacteria, the empirical choice should be decided on the basis of local ecology. 3rd GC, third-generation cephalosporin; ARDS, acute respiratory distress syndrome; ATB, antibiotics; GNB, Gram-negative bacteria; MDR, multidrug-resistant; MRSA, methicillin-resistant *Staphylococcus aureus*; PA, *Pseudomonas aeruginosa*; PIP/TAZ, piperacillin-tazobactam; R, Resistant; VAP, ventilator-associated pneumonia.

The initial treatment of VAP is based on empirical choices; however, an inappropriate initial antibiotic choice is associated with increased mortality ^21, 73^. In addition, the recovery of MDR bacteria is clearly associated with an increased risk of inappropriate therapy ^74^. As discussed earlier, the risk of MDR is conditioned by the local ecological data, previous colonization, and previous antibiotic therapy received by the patients. The increase in the risk of MDR in late-onset infections is challenged by recent studies. Regimens proposed by the North American guidelines are listed in Table 2 ^7^. An algorithm for an empirical therapy strategy combining guidelines and practical rules is proposed in Figure 3.

**Table 2.** Empirical treatment of hospital-acquired pneumonia/ventilator-associated pneumonia.

Not at high risk of mortality and no risk factors ^a^	Not at high risk of mortality but with factors increasing the likelihood of Gram-negative bacteria	High risk of mortality or receipt of intravenous antibiotics during the prior 90 days
One of the following: Piperacillin-tazobactam 4.5 g IV q6h OR Cefepime 2 g IV q8h Levofloxacin 750 mg IV daily	Piperacillin-tazobactam 4.5 g IV q6h OR Cefepime or ceftazidime 2 g IV q8h OR Levofloxacin 750 mg IV daily Ciprofloxacin 400 mg IV q8h OR Imipenem 1g IV q8h Meropenem 1 g IV q6h	Piperacillin-tazobactam 4.5 g IV q6h OR Cefepime or ceftazidime 2 g IV q8h OR Levofloxacin 750 mg IV daily Ciprofloxacin 400 mg IV q8h OR Imipenem 1g IV q8h Meropenem 1 g IV q6h AND Amikacin 25 (30) mg/kg IV daily OR Gentamicin 5–7 mg/kg IV daily OR Tobramycin 5–7 mg/kg IV daily
	Vancomycin 15 mg/kg IV q8–12h with goal to target 15–20 mg/mL trough level (consider a loading dose of 25–30 mg/kg × 1 for severe illness) OR Linezolid 600 mg IV q12h	Vancomycin 15 mg/kg IV q8–12h with goal to target 15–20 mg/mL trough level (consider a loading dose of 25–30 mg/kg × 1 for severe illness) OR Linezolid 600 mg IV q12h

Adapted from Infectious Diseases Society of America/American Thoracic Society guidelines ^7^. ^a^Risk factors of multidrug-resistant ventilator-associated pneumonia (VAP) are prior intravenous use within 90 days, septic shock at VAP onset, acute respiratory distress syndrome preceding VAP, five or more days of hospitalization prior to VAP onset, and acute renal replacement therapy prior to VAP onset. IV, intravenous; q, every.

The challenge for the intensivist is to start an antimicrobial therapy that will be immediately effective while avoiding any overuse of extended-spectrum antimicrobials. New rapid diagnostic tests have been developed but their performances for VAP diagnosis remain to be evaluated ^75, 76^. Rapid nucleic acid amplification or mass spectrometry-based techniques provide rapid identification of targeted microorganisms. Some of these new tests are also able to detect resistance genes. However, the presence of genes detected by these techniques does not mean that the pathogens are alive or dead, nor does it provide information regarding phenotypic antimicrobial susceptibility. Rapid culture with semi-automated rapid antibiotic susceptibility tests are also in development. Fluorescence *in situ* hybridization-based microscopy identification and antibiotic susceptibility test (ID/AST) systems can evaluate antibiotic susceptibility from respiratory secretions on a previously defined panel of pathogens. A recent pilot study reported promising results: the technique was able to detect pathogens in bronchoalveolar lavage after 5 ± 7 hours of culture and 5 hours of analysis, and sensitivity and specificity were 100% and 97%, respectively ^77^. Technical developments with a better selection and quantification of pathogens and resistance patterns are warranted.

Beta-lactams remain a cornerstone antibiotic for the treatment of VAP. Critical care patients exhibit high clearance and distribution volume, which contribute to low blood levels of antimicrobials ^78^. Therefore, the doses that should be used to treat the most severe patients are frequently higher than the ones approved by regulatory agencies ^79^. For β-lactams, the best results seem to be associated with β-lactam plasma levels up to four times the minimal inhibitory concentration (MIC) of the involved pathogen and during 100% of the interval between each dose ^80^. For these agents, a loading dose followed by a continuous infusion may be a relevant method of administration to increase the antibiotic concentration in the blood and the lung lining fluid with a lower risk of neurological ^81^ or renal ^82^ toxicity. It is of special importance when bacteria are not fully susceptible or when MICs are high, as for *P. aeruginosa* and MDR Gram-negative bacteria ^83^.

Combination therapy with aminoglycosides increases the likelihood to immediately achieve an adequate therapy, especially for infection due to MDR Gram-negative bacteria ^84^. It is associated with an improved prognosis in the most severe patients ^85^. A dose as high as 25 mg/kg of amikacin is required to reach the optimal 60 mg/L peak concentration, even in the case of renal failure. Indeed, the distribution volume of aminoglycosides is not affected by renal dysfunction ^86^. However, renal impairment, present in almost 30% of ICU patients, will lead to prolonged intervals between doses, reducing the actual number of peak levels, thus possibly affecting the treatment efficiency.

Indeed, controversies still exist about advantages and disadvantages of aminoglycosides. Ong *et al*. compared empirical therapy of septic shock with or without gentamicin in two Dutch ICUs ^87^. One of the ICUs preferentially used aminoglycosides, whereas the other preferentially avoided them. After careful adjustment on patients’ characteristics, they found that gentamicin is an independent predictor of renal failure without affecting mortality. The main limitations of this study include (1) the absence of random allocation, (2) the absence of control on center effect, (3) the setting in two ICUs where the rate of MDR Gram-negative organisms is very low with a minimal risk of inadequate therapy, and (4) the absence of therapeutic drug monitoring for gentamicin. Nevertheless, the study by Ong *et al*. ^87^ emphasizes the importance of new well-conducted studies comparing antimicrobial therapy with or without aminoglycosides, given with careful therapeutic drug monitoring, for the most severe ICU patients.

Combination therapy with fluoroquinolones was associated with a decrease in the number of days alive without relapse or reinfection in a cohort of patients with VAP due to *P. aeruginosa* or *Enterobacteriaceae* and without any prior treatment with fluoroquinolones ^88^. However, the fluoroquinolones are inconstantly active against MDR Gram-negative bacteria and their use is associated with an important risk of emergence of MDR bacteria in the lung and gut microbiota ^89^.

Concerning extended-spectrum β-lactamase-producing *Enterobacteriaceae* (ESBL-PE), carbapenems remain the first-line agents despite leading to the risk of emergence and spread of carbapenemase-producing *Enterobacteriaceae* ^90^. Other options such as piperacillin/tazobactam or a high dose of third-generation cephalosporins administered by continuous infusion could be considered, especially as step-down therapy for ESBL-PE with low MICs ^30^. New compounds such as ceftolozane/tazobactam and ceftazidime/avibactam have recently been released; however, against HAP/VAP, only the latter was non-inferior to meropenem in a recent randomized trial (REPROVE NCT01808092; results available on ClinicalTrials.gov, not yet published). Temocillin, a ticarcillin derivative that resists ESBL, can be used but only as a step-down therapy for pathogens with MICs below 8 mg/L ^91^. For carbapenem-resistant Gram-negative bacteria, colistin is a cornerstone of the treatment, although ceftazidime/avibactam association might be effective. A new association of meropenem and vaborbactam (M-V), recently approved for severe urinary tract infections in the US, has been compared with best available therapy (BAT) in severe infection presumably due to carbapenemase R *Enterobacteriaceae*, including nosocomial pneumonia (TANGO-2 NCT 02168946). The study was presented at the IDWeek convention (in San Diego, CA, in October 2017; abstract 1867) and enrolled 43 patients (more than 80% with *Klebsiella pneumoniae carbapenemase*). It has been stopped prematurely for significant superiority in terms of clinical failure, nephrotoxicity, and non-significant improvement of day-28 mortality of patients with HAP/VAP (M-V 4/16 versus BAT 4/9). Further studies are awaited to confirm these encouraging results and make formal recommendations on its use. An intravenous colistin regimen should be considered with a loading dose of 9 MU and with caution regarding its potential nephrotoxicity ^92^.

Inhaled antimicrobial therapy may be considered, as this route of administration enables very high concentrations of antimicrobials to be locally delivered ^93, 94^. However, there are no solutions specifically formulated for inhalation, and a limited number of devices are designed for the nebulization of antibiotics ^95, 96^. Of note, despite the possible advantages in terms of microbiological eradication and emergence of resistance ^97^, no impact on patient prognosis has been demonstrated ^94, 97– 99^.

In units with rates of methicillin-resistant *Staphylococcus aureus* (MRSA) around 10–20%, include vancomycin or linezolid in the empirical therapy ^7^. When the MIC to vancomycin is higher than 1.5 mg/L, the mortality of MRSA pneumonia is higher ^100^. Moreover, it is very difficult to reach pharmacokinetic targets using vancomycin ^101^ without any increase in renal toxicity. Consequently, linezolid should be preferred, particularly in patients with renal impairment or if the MRSA MIC to vancomycin is over 1.5 mg/L ^102^.

An 8-day antibiotic course appears safe in VAP. This duration can be shortened when a procalcitonin-guided algorithm is used ^103^ or when ventilator settings (PEEP ≤5 cm H _2_O and FiO _2_ ≤40%) are stable for 48 hours after antibiotic initiation ^104^. As procalcitonin levels above 1.5 ng/mL after three days of treatment seemed strongly associated with a poor outcome ^105^, re-evaluation of the accuracy of diagnosis and a search for drainable collections (for example, lung abscess or empyema) and revision of therapeutic antimicrobial regimens should be promptly revisited when procalcitonin levels remain high. However, definite data are lacking where *Pseudomonas*, *Acinetobacter*, *Stenotrophomonas*, and MRSA are concerned.

## Abbreviations

BAT, best available therapy; CDC, Centers for Disease Control and Prevention; ESBL-PE, extended-spectrum beta-lactamase-producing *Enterobacteriaceae*; FiO _2_, fraction of inspired oxygen; HAP, hospital-acquired pneumonia; ICU, intensive care unit; IVAC, infection-related ventilator-associated complication; MDR, multidrug-resistant; MIC, minimal inhibitory concentration; MRSA, methicillin-resistant *Staphylococcus aureus*; M-V, meropenem and vaborbactam; OR, odds ratio; PEEP, positive end expiratory pressure; SDD, selective digestive decontamination; SOD, selective oropharyngeal decontamination; VAC, ventilator-associated complication; VAE, ventilator-associated event; VAP, ventilator-associated pneumonia; VAT, ventilator-associated tracheobronchitis.
